# New Model for Osteoporosis Risk Screening Using Emergency Department Visits

**DOI:** 10.7759/cureus.22237

**Published:** 2022-02-15

**Authors:** Andrew Alabd, Andre Alabd, Matthew Miller, Carrie Walsh, Alex Silverman, Nooreen Dabbish, Chol Kuoiloi, Stanton Miller

**Affiliations:** 1 Department of Medicine, Cooper University Hospital, Camden, USA; 2 Department of Urology, Indiana University Health, Indianapolis, USA; 3 Emergency Medicine, Thomas Jefferson University Hospital, Philadelphia, USA; 4 Emergency Medicine, Massachusetts General Hospital, Harvard Medical School, Boston, USA; 5 Statistics, Thomas Jefferson University Hospital, Philadelphia, USA; 6 Research, Thomas Jefferson University Hospital, Philadelphia, USA

**Keywords:** fracture, modifiable risk factors, health maintenance, screening, osteoporosis

## Abstract

Background

Even though osteoporosis is the most common bone disease in the United States, it is frequently underscreened and underdiagnosed. In this study, we aimed to utilize the Emergency Department to conduct preemptive osteoporosis risk screening and assess the risk associated with gender and race based on a statistical analysis of survey responses.

Methodology

Patients >40 years of age presenting at two Emergency Departments were eligible. Consenting patients were asked questions from a modified *One-Minute Osteoporosis Risk Test*. Modifiable, fixed, and total (modifiable risks + fixed risks) risk sums were calculated. For the association test, chi-square and Wilcoxon rank-sum tests were used. Four total risk categories were created (0-1, 2-3, 4-5, 6+). Odds of being in a higher risk category were analyzed using univariate ordinal logistic regression.

Results

The prevalence of both a fixed and modifiable risk was 62.2%. Women were more likely than men to report a risk (81.2% vs. 67.5%; p = 0.0043) and to be in a higher risk category (odds ratio (OR) [95% confidence interval (CI)] = 1.63 [1.09-2.45]; p = 0.018). Evidence strongly indicated an unadjusted association of race and modifiable risk category (p < 0.001), with more than half of African Americans (53.0%) in the highest category compared to 26.0% of whites. The total risk was higher in African Americans than whites (OR [95% CI] = 1.75 [1.15-2.67]; p = 0.010).

Conclusions

Race and gender were associated with specific risk factors. The Emergency Department proved to be a feasible location for conducting health maintenance screenings and should be considered for patient-specific routine osteoporosis risk screenings.

## Introduction

Osteoporosis is a major public health concern in our aging population. Often asymptomatic until a bone fracture has occurred, osteoporosis is characterized by low bone mass, decreased bone strength, and increased risk of fractures [1,2]. Osteoporosis is especially prevalent in older adults, and fractures in this population are associated with psychological disorders, permanent disability, and an increased risk of mortality [3]. The sequelae of osteoporosis lead to a significant economic burden, with an estimated annual healthcare cost of $17 to $20 billion since 2005, a number that is expected to exceed $25 billion by the year 2025 [1,4,5].

Despite the growing burden of osteoporosis, it remains largely underdiagnosed and undertreated, especially among racial and ethnic minority groups in the United States [1,6-8]. Previously published literature has described 20-30% treatment rates for women and men even after diagnosis [9]. This discrepancy may reflect several barriers to treatment, including an inadequate understanding of the risks of osteoporosis among patients and physicians, lack of access to diagnostic tests, and cost of therapy [10-12]. For all of these reasons, there is a growing interest in improving preemptive osteoporosis screening. Some tools for estimating osteoporosis-related fracture risk include the World Health Organization Fracture Risk Assessment Tool and the Foundation for Osteoporosis Research and Education 10-Year Fracture Risk Calculator. The International Osteoporosis Foundation (IOF) has developed a *One-Minute Osteoporosis Risk Test*. This survey is an internationally recognized awareness-raising tool, which is composed of 19 questions intended to help patients assess their own risk for osteoporosis and pursue further evaluation by their primary care physician [13,14]. While previous studies employing this survey have been conducted in community centers, outpatient clinics, and non-acute care settings, this study was conducted in the Emergency Department (ED) [13-17].

Currently, limited research exists on the utility of the ED as a location for an osteoporosis screening program for older non-fractured adults, although the need for preventive measures as part of ED care has been explored in previous studies [18,19]. Because osteoporosis is a preventable disease and a significant public health issue, especially considering the growing aging population, the ED can serve a vital role in identifying patients at risk for osteoporosis through preventive screening. Two studies with a similar aim have been published to screen non-fracture patients for osteoporosis in an ED setting [20,21]. One study required a low-energy fall for patient eligibility and incorporated osteodensitometry as part of the screening process. The other study required a calcaneus ultrasound (US) as part of the screening process. However, our study differed because it screened all patients aged 40 years or older who presented to the ED using a brief survey to increase the feasibility of its administration to a large patient population.

This study aimed to explore the feasibility of utilizing patient wait time in an ED setting to screen for osteoporosis risk. To our knowledge, this study is the first to use the IOF *One-Minute* test to screen for osteoporosis in patients aged 40 and above in the ED. By adding four questions to the standard IOF *One-Minute* test, we assessed possible increased risks associated with race and gender. Given the significant impact of osteoporosis, the ever-increasing number of individuals at risk, and the need for conducting mass osteoporosis screening in one setting, the ED can serve as a model for maximizing health maintenance for diverse patients.

## Materials and methods

Study population

The study was approved by the Office of Human Research at Jefferson (approval number: 15E.397). The target population included patients aged 40 years or older who presented to the EDs of two urban hospitals in southeastern Pennsylvania from February 1, 2016, to November 17, 2016. Age 40 was chosen as the minimum age requirement for questionnaire eligibility as a reflection of entry into a middle-aged population. Eligible patients were identified from the roster at each ED and approached by study personnel only if they appeared to be waiting for treatment. Trained study personnel, consisting of medical students and post-baccalaureate students, obtained consent and administered the questionnaire to each patient in person. The total time spent with each patient was approximately 20 minutes. The study personnel was not blind to the study hypothesis. Consent was documented through a consent form signed by the patient and the research associate. The study was approved by the Institutional Review Board before gathering data.

Measures

Patients were asked questions from the IOF *One-Minute Osteoporosis Risk Test* [22]. The original questionnaire consisted of 19 questions and we added four questions asking for patients’ race/ethnicity, age, gender, and zip code. These aimed at identifying patients at high risk of osteoporosis based on age, gender, ethnicity, or geographical location. The questionnaire also assessed parent history and modifiable risk questions. Patients were not asked specifically about their osteoporosis status. Risk factors assessed by the questionnaire included race, gender, age, medical history, family history, and modifiable habits.

Statistical analysis

All participants who completed the full questionnaire were included. Modifiable risk (scale 0-5), fixed (0-11), and total risk (0-16) sums were calculated. Five modifiable risk questions were summed. Fixed risk sums consisted of seven history, two parental history, and two gender/age-specific questions. The total risk sum was fixed risk plus modifiable risk. Association tests for risk factors and categorical variables (i.e., race, gender) were performed using chi-square tests. Association tests for non-parametrically distributed continuous variables (i.e., risk sum) were performed using the Wilcoxon rank-sum test. Total risk categories were created based on 0-1, 2-3, 4-5, and 6+ risk factors. Modified risks categories were none, one, and two or more risks. Fixed risk categories were none, one, one to two, and three or more risks. Univariate ordinal logistic regression was conducted to evaluate the odds of a higher risk category. A backwards, stepwise selection procedure was used to build all multiple variable logistic models. Hypothesized importance and univariate association (p-values of <0.2) were used for entry, followed by sequential elimination of covariates with adjusted association (p-values <0.2) repeated until all independent variables met these criteria. All statistical analyses were performed in SAS version 9.4 (SAS Institute, Cary, NC, USA).

## Results

The questionnaire was completed by 341 patients over a nine-month period, a sample size estimated to achieve significant statistical power. The demographic data of interviewed patients are shown in Table 1. Most patients were female (64%), and the mean age was 61.3 years. More than half of the patients identified themselves as white (57.6%), and approximately one-third identified as African American (33.7%). Overall, 125 (36.7%) patients were in the highest modifiable risk category, and 76 (22.3%) patients were in the fixed risk category. A total of 311 (91.2%) patients reported having at least one risk factor, and 212 (62.2%) patients reported having both at least one fixed and one modifiable risk factor (Table 2). Increased age was associated with increased odds for higher fixed risk (10-year odds ratio (OR) = 1.57 [1.33-1.86]; p < 0.001) and for higher total risk (10-year OR = 1.43 [1.22-1.67]; p < 0.001).

**Table 1:** Demographic data of the study participants.

Demographic	N = 341	(Col %)
Gender		
Male	123	(36.1)
Female	218	(63.9)
Age, mean (SD)	61.3 (12.8)	-
Race		
White	196	(57.5)
African American or Black	115	(33.7)
Other	30	(8.8)
Distance from questionnaire location (in 100-mile units), median (minimum, maximum)	0.038 (0, 24)	-

**Table 2:** Fixed, modifiable, and total risk category population distribution.

Risk categories (questions included)	N = 341	(Col %)
Fixed risk category		
None	83	(24.3)
1-2 factors	182	(53.4)
3 or more factors	76	(22.3)
Modifiable risk category		
None	73	(21.4)
1-2 factors	143	(41.9)
3 or more factors	125	(36.7)
Total risk category		
None or 1 factor	81	(23.8)
2-3 factors	136	(39.9)
4-5 factors	95	(27.9)
6 or more factors	29	(8.5)

The data suggest that the female gender was associated with a higher incidence of frequent falls. The female gender was also associated with low calcium intake and reduced physical activity. In addition to this observed increase in modifiable risk, women also had an increase in fixed risk (Table 3). Females were more likely than males to be in a higher total risk category (OR = 1.63 [1.09-2.45]; p = 0.018) and more likely to report a risk (81.2% vs. 67.5%; p = 0.0043).

**Table 3:** Risk factor categories by gender.

Risk categories (questions included)	Female (Col %)	Male (Col %)	P-value
Fixed risk category			<0.001
None	39 (17.89)	44 (35.77)	
1-2 factors	122 (55.96)	60 (48.78)	
3 or more factors	57 (26.15)	19 (15.45)	
Modifiable risk category			0.382
None	49 (22.48)	24 (19.51)	
1-2 factors	95 (43.58)	48 (39.02)	
3 or more factors	74 (33.94)	51 (41.46)	
Fixed and modifiable risk category			0.042
None or 1 factor	41 (18.81)	40 (32.52)	
2-3 factors	92 (42.2)	44 (35.77)	
4-5 factors	65 (29.82)	30 (24.39)	
6 or more factors	20 (9.17)	9 (7.32)	

African American patients (Table 4) were more likely than white patients to report that they had been diagnosed with rheumatoid arthritis (p = 0.028), that they participated in less than 30 minutes of daily physical activity (p = 0.0049), and that they were getting less than 10 minutes of sun exposure without taking vitamin D supplements (p = 0.0012). Compared to white patients, African American patients had more modifiable risks for osteoporosis (unadjusted OR = 2.65 [1.70-4.14]; p < 0.001; overall race, p < 0.001). African Americans also had higher odds of an increased total risk category (OR = 1.75 [1.15-2.67]; p = 0.010; overall race comparison, p = 0.033).

**Table 4:** Risk factor categories by race. OR: odds ratio; CI: confidence interval

Risk categories (questions included)	White OR (95% CI) N (%)	African American, or Black OR (95% CI) N (%)	Other OR (95% CI) N (%)	P-value
Fixed risk category	Ref.	1.01 (0.65-1.58; p = 0.466)	1.31 (0.63-2.74; p = 0.949)	0.763
None	50 (25.51)	6 (20)	27 (23.48)	
1-2 factors	102 (52.04)	16 (53.33)	64 (55.65)	
3 or more factors	44 (22.45)	8 (26.67)	24 (20.87)	
Modifiable risk category	Ref.	2.65 (1.70-4.14; p < 0.001>	1.39 (0.68-2.85; p = 0.366)	<0.001>
None	47 (23.98)	9 (30)	17 (14.78)	
1-2 factors	98 (50)	8 (26.67)	37 (32.17)	
3 or more factors	51 (26.02)	13 (43.33)	61 (53.04)	
Total risk category	Ref.	1.75 (1.15-2.67; p = 0.010)	1.39 (0.69-2.81; p = 0.357)	0.033
None or 1 factor	54 (27.55)	7 (23.33)	20 (17.39)	
2-3 factors	80 (40.82)	11 (36.67)	45 (39.13)	
4-5 factors	50 (25.51)	9 (30)	36 (31.3)	
6 or more factors	12 (6.12)	3 (10)	14 (12.17)	

Multivariate models suggested that age, gender, and physical activity were the principal predictors for individual risk of osteoporosis. For the entire population, as well as females only, there were no significant predictors of total modifiable risks in the adjusted model. Adjusted age (10-year OR = 1.22 [1.05-1.43]; p = 0.041) and gender (OR = 1.23 [1.01-1.51]; p = 0.041) were associated with increased total risks. Adjusted increases in age (10-year OR = 1.27 [1.08-1.50]; p = 0.004) and low physical activity (OR = 1.58 [1.00-2.51]; p = 0.051) were associated with higher fixed risks among all patients.

## Discussion

The results of our study indicate that the ED is an effective setting for initiating osteoporosis screening among all patients aged 40 and older. The data that we collected from the questionnaire (Table 5 in Appendices) indicate that many adults over the age of 40 have at least one risk factor for osteoporosis and that women and African Americans are particularly likely to have multiple risk factors. The increased risks among female participants can be attributed to specific biological factors, such as a decrease in estrogen after menopause, and modifiable risk factors, such as reduced exercise and low calcium intake. These findings reinforce previous research that the prevalence of osteoporosis is considerably higher in postmenopausal women than in older men [23].

The data demonstrated that African American women have a further increased total risk for osteoporosis than white women. Additionally, African American participants reported the highest percentage of modified risk compared to all other participants. The increased odds can be attributed to specific biological risk factors and modifiable risk factors, such as reduced physical activity. In particular, African American patients were more likely to report having less than 10 minutes of daily exposure to sunlight without taking vitamin D supplements. Both the lack of sun exposure and the absence of vitamin D supplement intake serve as strong indicators of a higher modified risk for osteoporosis because they are needed to modulate the action of various hormones that keep bone resorption and formation in check. Although vitamin D insufficiency is more prevalent among African Americans because their pigmentation reduces vitamin D production in the skin, they have lower rates of osteoporotic fractures, which may be explained by bone-protective adaptations and skeletal resistance to the parathyroid hormone [24,25].

Therefore, our findings reinforced prior findings that African Americans have higher modifiable risks but lower fixed risks compared to whites. Determining the primary biological or behavioral factors that contribute to racial differences in osteoporosis risk remains unknown. These findings suggest that preventive care is needed to identify more patients with increased modifiable risk. Our findings also indicate that the ED can serve as a viable setting for mass screening a diverse group of patients, especially populations most at risk. In particular, wellness screenings in the ED can help fulfill the need for improved prevention and earlier diagnosis of osteoporosis.

Our findings indicate that the ED provides an appropriate and opportune setting to implement these interventions. Patients who are screened in the ED and determined to have a moderate-to-high risk for osteoporosis can be provided educational materials on risk factor modification and referred to the appropriate provider upon discharge. Such referrals can immediately be expedited through electronic health records. Therefore, patient health maintenance screenings in the ED can ultimately improve clinical outcomes. An additional benefit of screening programs in the ED may be their ability to reduce patient perception of wait times. Previous interventions have demonstrated that decreased perception of wait times in the ED have resulted in improved overall patient satisfaction [26].

Future efforts should focus on developing novel interventions, such as the model proposed in this study, to improve patient satisfaction and clinical outcomes. Future directions from this project include making use of electronic health records to record screening results, track patients, and ensure proper compliance with recommendations after discharge by utilizing electronic health records to expedite referral to a primary care provider. Ultimately, this model of care can lead to the introduction and implementation of a culturally specific screening curriculum within the hospital’s ED and primary care practice to capture and track the longitudinal progress of all patients with osteoporosis risk.

Our sample size depended on patients in the ED at the time we administered the survey. In addition, study personnel did not record the number of patients who declined to participate in the survey. Therefore, our sample may not be fully representative of all patients in the ED. Additionally, because the surveyed patients were waiting in the ED to receive treatment, they may have been more likely to be forgetful or to feel rushed while answering the survey. In future studies, including questions regarding patient knowledge of osteoporosis can yield data beneficial to the creation and implementation of educational intervention programs.

## Conclusions

This study indicates the utility of the ED for mass screening of osteoporosis risk by utilizing downtime to administer osteoporosis screening. Patient health maintenance screening in the ED can improve clinical outcomes. Ultimately, this model of care can lead to the introduction and implementation of a culturally specific screening curriculum within the hospital’s ED and primary care practice to capture and track the longitudinal progress of all patients with osteoporosis risk. This study confirmed gender and race disparities regarding osteoporosis risk and reinforced prior findings that African Americans have higher modifiable risks but lower fixed risks compared to whites. Further research should consider models of multifaceted clinical intervention for high-risk patients.

## Appendices

**Table 5:** Osteoporosis risk questionnaire.

Covariate	Question
Additional Q1	What is your biological gender?
Additional Q2	What is your age (in years)?
Additional Q3	What is your zip code?
Additional Q4	What is your race and/or ethnicity?
History Q1	Were you diagnosed with a bone fracture during your ER visit today?
History Q2	Do you fall frequently (more than once in the last year), or do you have a fear of falling because you are frail?
History Q3	After the age of 40, have you lost more than 3 cm in height (just over 1 inch)?
History Q4	Are you underweight (is your body mass index less than 19 kg/m^2^)?
History Q5	Have you ever taken corticosteroid tablets (cortisone, prednisone, etc.) for more than three consecutive months?
History Q6	Have you ever been diagnosed with rheumatoid arthritis?
History Q7	Have you been diagnosed with an over-active thyroid, over-active parathyroid glands, type 1 diabetes, or a nutritional/gastrointestinal disorder such as Crohn’s or Celiac disease?
Modifiable risk Q1	Do you regularly drink alcohol in excess of safe drinking limits (more than two units a day)?
Modifiable risk Q2	Do you currently, or have you ever, smoked cigarettes?
Modifiable risk Q3	Is your daily level of physical activity less than 30 minutes per day (housework, gardening, walking, running, etc.)?
Modifiable risk Q4	Do you avoid, or are you allergic to milk or dairy products, without taking any calcium supplements?
Modifiable risk Q5	Do you spend less than 10 minutes per day outdoors with part of your body exposed to sunlight), without taking vitamin D supplements?
History men Q1	Have you ever suffered from impotence, lack of libido, or other symptoms related to low testosterone levels?
History parent Q	Have either of your parents been diagnosed with osteoporosis or broken a bone after a minor fall (a fall from standing height or less)?
History parent Q2	Did either of your parents have a stooped back (dowager’s hump)?
History women Q1	For women over 45: Did your menopause occur before the age of 45?
History women Q2	Have your periods ever stopped for 12 consecutive months or more (other than because of pregnancy, menopause, or hysterectomy)?
History women Q3	Were your ovaries removed before age 50, without you taking hormone replacement therapy?
Adapted from international osteoporosis foundation IOF One-Minute test.	
